# Genome-Wide Profiling and Phylogenetic Analysis of the *SWEET* Sugar Transporter Gene Family in Walnut and Their Lack of Responsiveness to *Xanthomonas arboricola* pv. *juglandis* Infection

**DOI:** 10.3390/ijms21041251

**Published:** 2020-02-13

**Authors:** Shijiao Jiang, Bipin Balan, Renata de A. B. Assis, Cintia H. D. Sagawa, Xueqin Wan, Shan Han, Le Wang, Lanlan Zhang, Paulo A. Zaini, Sriema L. Walawage, Aaron Jacobson, Steven H. Lee, Leandro M. Moreira, Charles A. Leslie, Abhaya M. Dandekar

**Affiliations:** 1 Department of Plant Sciences, University of California, Davis, CA 95616, USA; Jiangshijiao@outlook.com (S.J.); bipinbioinfo@gmail.com (B.B.); redab@ucdavis.edu (R.d.A.B.A.); chdsagawa@ucdavis.edu (C.H.D.S.); lerwang@ucdavis.edu (L.W.); zlanapple@zafu.edu.cn (L.Z.); pazaini@ucdavis.edu (P.A.Z.); slwalawage@ucdavis.edu (S.L.W.); ajacobson@ucdavis.edu (A.J.); shle@ucdavis.edu (S.H.L.); caleslie@ucdavis.edu (C.A.L.); 2 College of Life Sciences, China West Normal University, Nanchong 637000, China; 3 Dipartimento di Scienze Agrarie Alimentari Forestali, Università di Palermo, Viale delle Scienze Ed. 4, 90128 Palermo, Italy; 4 Departamento de Ciências Biológicas, Instituto de Ciências Exatas e Biológicas, Núcleo de Pesquisas em Ciências Biológicas, Universidade Federal de Ouro Preto, Ouro Preto 35400-000, Brazil; lmmorei@gmail.com; 5 Department of Forestry, Sichuan Agricultural University, Chengdu 611130, China; w-xue@163.com (X.W.); hanshan6618@163.com (S.H.); 6 Department of Horticulture, College of Agriculture and Food Science, Zhejiang A&F University, Lin’an, Hangzhou 311300, China

**Keywords:** SWEET sugar transporters, gene family, phylogeny, TAL effector, gene expression, walnut blight, *Xanthomonas*

## Abstract

Following photosynthesis, sucrose is translocated to sink organs, where it provides the primary source of carbon and energy to sustain plant growth and development. Sugar transporters from the SWEET (sugar will eventually be exported transporter) family are rate-limiting factors that mediate sucrose transport across concentration gradients, sustain yields, and participate in reproductive development, plant senescence, stress responses, as well as support plant–pathogen interaction, the focus of this study. We identified 25 *SWEET* genes in the walnut genome and distinguished each by its individual gene structure and pattern of expression in different walnut tissues. Their chromosomal locations, *cis*-acting motifs within their 5′ regulatory elements, and phylogenetic relationship patterns provided the first comprehensive analysis of the *SWEET* gene family of sugar transporters in walnut. This family is divided into four clades, the analysis of which suggests duplication and expansion of the SWEET gene family in *Juglans regia*. In addition, tissue-specific gene expression signatures suggest diverse possible functions for *JrSWEET* genes. Although these are commonly used by pathogens to harness sugar products from their plant hosts, little was known about their role during *Xanthomonas* *arboricola* pv. *juglandis* (*Xaj*) infection. We monitored the expression profiles of the *JrSWEET* genes in different tissues of “Chandler” walnuts when challenged with pathogen *Xaj*417 and concluded that SWEET-mediated sugar translocation from the host is not a trigger for walnut blight disease development. This may be directly related to the absence of type III secretion system-dependent transcription activator-like effectors (TALEs) in *Xaj*417, which suggests different strategies are employed by this pathogen to promote susceptibility to this major aboveground disease of walnuts.

## 1. Introduction

Following photosynthesis, sugars like sucrose, transported from their photosynthetic “sources” to heterotrophic “sinks”, enable plant growth and development [1,2,3]. Sucrose is the predominant form of sugar translocated in many plant species and is driven by hydrostatic pressure generated in the phloem to mediate long-distance distribution throughout the plant [4,5,6]. However, sucrose transport across the plant cell membrane requires the assistance of appropriate sugar transporters. A proton-coupled sucrose transporter (SUT) that acts as a sugar/H^+^ symporter is essential for translocation [7], while a sieve element–companion cell complex (SE/CC) is required for translocation into the phloem. Another type of sugar transporter known as SWEETs (sugar will eventually be exported transporters) effuse sucrose from inside the cell into the cell wall as a prerequisite for SUT1-mediated phloem loading [1]. Since these sugar transporters regulate the sucrose pools in plants, several microorganisms vie for carbohydrates from plants through this pathway. Increasing evidence suggests that both bacterial and fungal pathogens hijack sugar transporters to gain access to pools of sucrose for growth [8,9,10]. Unlike SUTs, which are expressed at a low level, require energy, and are therefore a rate-limiting factor for sucrose transport, SWEET proteins function as key transporters for sucrose, hexose, and fructose along a concentration gradient and become a priority target in plant–pathogen interactions [11,12,13].

The SWEET protein family members typically contain seven transmembrane domains (TMDs) harboring two MtN3/saliva domains, also referred to as the PQ-loop-repeat (PFAM code PF0383). This family was first identified with 17 members in *Arabidopsis* and 21 in rice [1,14,15]. Phylogenetic analysis revealed that SWEET proteins can be divided into four clades according to the type of substrate transported. Clades I, II, and IV members are monosaccharide efflux transporters, of which clades I and II are located on the plasma membrane and preferentially transport hexoses, while characterized SWEETs from clade IV are fructose transporters located on the vacuolar membrane [1,16,17]. Clade III SWEETs are sucrose efflux transporters located on the plasma membrane. The genes that encode these transporters in rice are targeted by transcription activator-like effectors (TALEs) from *Xanthomonas oryzae* pv. *oryzae* (*Xoo*), such as seen in the interaction of PthXo1 TALE with *cis*-acting elements within the 5′ regulatory region of *OsSWEET11* and of PthXo2 with *OsSWEET13* [18,19]. The pathogen *Xoo* can upregulate expression of the corresponding *SWEET* genes effectively, recruiting a specific SWEET protein to deliver sucrose to the apoplast and provide the bacteria with a carbon source [20,21,22,23]. Furthermore, clade III SWEET proteins GhSWEET10 in cotton and MeSWEET10a in cassava are also activated by TALEs from *X. citri* subsp. *malvacearum* (*Xcm*) and *X*. *axonopodis* pv. *manihotis* (*Xam*), respectively [24,25]. Notably, CsSWEET1 is the only SWEET protein from a different clade (clade I) that can be recruited by *X. citri* subsp. *citri* (*Xcc*), but in a TALE-dependent manner when connected directly to the target gene *LOB1* [26]. Other SWEET proteins, including SWEETs in other clades, can be induced during plant interaction with fungi, symbiotic bacteria, and oomycetes [9,23,27]. Host SWEET proteins are induced in many other systems such as the infection of *A. thaliana* by *Pseudomonas syringae* pv. *tomato* DC3000, and by the obligate biotrophic powdery mildew pathogen *Golovinomyces cichoracearum* [28], the infection of grapes with *Botrytis cinerea* [29], *Medicago truncatula* with *Sinorhizobium meliloti* [30], oilseed rape by *Sclerotinia sclerotiorum* [31], wheat by *Puccinia* [32], banana by *Fusarium oxysporum* f. sp. *cubense* TR4 [33], and cabbage by *Plasmodiophora brassicae* [34]. It is thus reasonable to speculate that SWEETs play a pivotal role in plant–pathogen interactions and may serve as host susceptibility factors for many pathogens.

**Figure 1:**
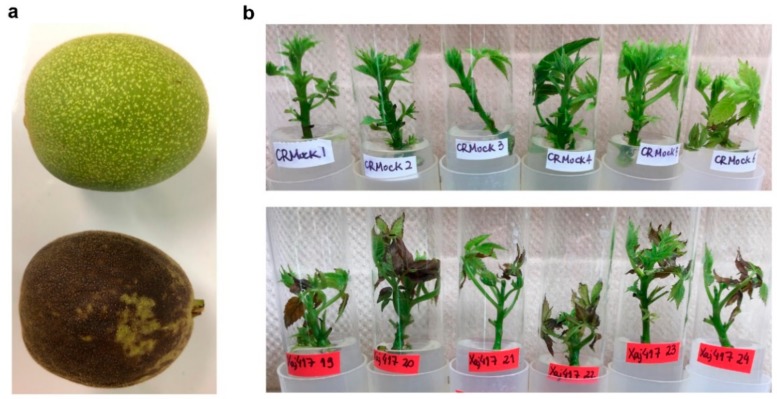
Bacterial walnut blight symptoms. (**a**) A healthy nut still with hull tissue is shown on top, contrasted with a walnut blight symptomatic nut on the bottom. (**b**) Blight symptoms can also be observed in microshoots maintained in tissue culture under controlled laboratory conditions. Top panel shows mock-inoculated shoots and bottom panel shows *Xaj*417-infected shoots four days post inoculation.

Walnut blight is the most serious aboveground disease in *Juglans regia* and is caused by the bacterial pathogen *X. arboricola* pv. *juglandis* (*Xaj*) (Figure 1). Since little is known about this plant–pathogen interaction, the current study examined the significance of *SWEET* genes as possible susceptibility genes, as occurs in rice bacterial leaf blight and other diseases also caused by *Xanthomonas*. Pathogens from *Xanthomonas* species are well known for modulating plant immune responses by secretion of effector proteins directly inside host cells through a highly conserved protein secretion system (T3SS) [35]. Comparative sequence analysis of T3SS effectors from *Xanthomonas* spp. demonstrates that they are divided into conserved protein families such as members of YopJ/AvrRxv, a family of predicted cysteine proteases, and members of the AvrBs3/PthA transcription factors, also designated transcription activator-like effectors (TALEs). TALEs have a nuclear localization signal and modulate host gene expression by direct interaction with the promoter region of specific genes in the host [36]. While vascular pathogens like *X. campestris* and *oryzae* typically use TALEs to modulate host gene expression, less is known about mesophillic pathogens such as *X. arboricola* and *X. citri*, although exceptions like *Xcm* are known to also employ TALEs [24]. Based on previous examples of pathogen TALEs or other effector types modulating host *SWEET* expression, we hypothesized that this interaction may also occur during walnut blight. To determine whether there is a causal relationship between *JrSWEET* genes and walnut blight, we first identified the *SWEET* family in the *Juglans regia* genome and analyzed gene structures, conserved domains, and established phylogenetic relationships. Next, we performed a comprehensive analysis of *JrSWEET* gene expression patterns in different walnut tissues in response to infection with *Xaj* and their correlation with other biomarkers of infection, such as polyphenol oxidase (PPO), which is induced during the interaction between walnut and *Xaj* [37,38]. In addition, we searched for TALEs in the *Xaj* genome and attempted to predict the target *JrSWEET* genes that would be induced during controlled in vitro inoculations.

## 2. Results

### 2.1. Genome-Wide Identification of SWEET Family Genes in Walnut

Based on HMMER, 37 putative *SWEET* genes were matched with an E-value cutoff of 0.0001 using typical SWEET domains (PFAM: PF03083) as queries. Meanwhile, based on BLAST searches, 29 putative *SWEET* genes were identified using *AtSWEET* sequences as queries. Finally, 25 non-redundant *JrSWEET* genes were confirmed with conserved core domains and complete gene structure by KEGG, CDD, and SMART (Table 1). The homologous genes of *AtSWEETs* were used to name the corresponding *SWEET* genes in *J. regia*. The *JrSWEET* genes were named using numerals and small letters, followed by capital letters to distinguish paralogous genes on the basis of their position in chromosomal pseudomolecules.

The 25 predicted JrSWEET proteins range from 154 (JrSWEET16A) to 301 (JrSWEET4B) amino acid residues long, with molecular masses between 16.63 (JrSWEET16A) and 33.24 kDa (JrSWEET3A) and isoelectric points from 5.85 (JrSWEET17C) to 9.72 (JrSWEET16A). The predicted subcellular localizations of the *JrSWEET* genes are primarily the plasma membrane, but also include vacuolar membrane (JrSWEET2aB and JrSWEET17) and chloroplast membrane (JrSWEET17A) (Table 1). The highly variable characteristics of *JrSWEETs* suggest different functional roles in different biological processes.

**Figure 2:**
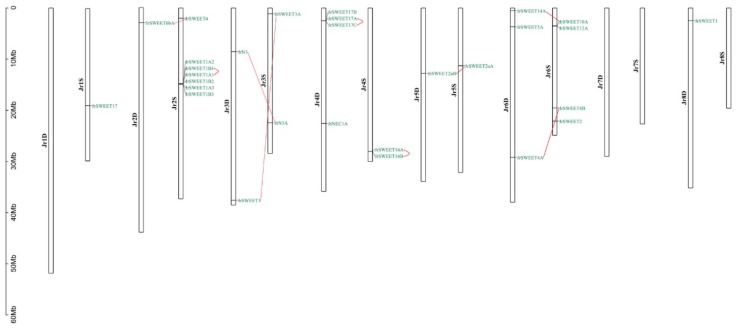
Positions of *SWEET* gene family members on the *Juglans regia* chromosomes. Duplicated genes are indicated with red lines.

The *JrSWEET* genes are distributed on 12 of 16 chromosomes; no *SWEET* genes were found on chromosomes *Jr1D*, *Jr7D*, *Jr7S*, or *Jr8S* (Figure 2). Five chromosomes (*Jr1S*, *2D*, *5D*, *5S*, and *8D*) harbored only a single gene, chromosomes *Jr3D*, *Jr3S*, and *Jr4S* had two genes each, and chromosome *Jr6D* had three genes. Chromosomes *Jr4D* and *Jr6S* had four genes each. Four groups of genes were linked closely on four chromosomes. Two pairs occurred on chromosomes *Jr4S* (*JrSWEET16A* and *16B*) and *Jr6S* (*JrSWEET10A* and *12A*). Chromosome *Jr4D* had three genes (*JrSWEET17A*, *17B*, and *17C*) and *Jr2S* had six genes (*JrSWEET1A1*, *1B1*, *1A2*, *1B2*, *1A3*, and *1B3*).

### 2.2. Gene Structure Analysis, Transmembrane Helix, and Motif Recognition of JrSWEETs

**Figure 3:**
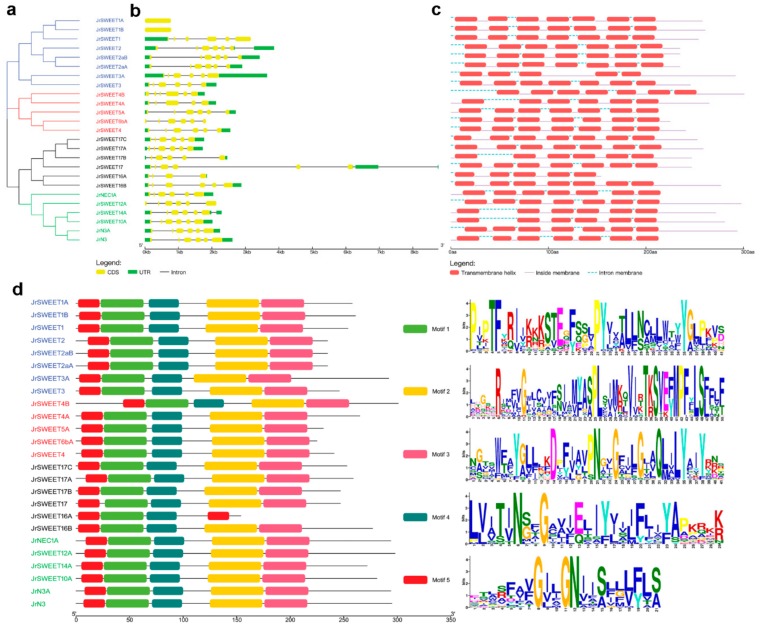
Phylogenetic relationship, gene structure, and distribution of conserved motifs of SWEET family proteins in *Juglans regia*. (**a**) The unrooted neighbor-joining phylogenetic tree of *SWEET* family members. Different clades are indicated by different colors: blue, clade I; red, clade II; green, clade III; and black, clade IV. (**b**) The exon/intron structures of 25 *SWEET* genes identified in walnut. Exons are represented by yellow boxes; introns by black lines; and upstream or downstream untranslated regions by green boxes. (**c**) The transmembrane helixes in JrSWEET proteins predicted by TMHMM. Red boxes: transmembrane helixes; purple lines: cytosol localization; and blue dashed lines: apoplast localization. (**d**) Conserved motifs in JrSWEET proteins. Five putative motifs are indicated in different colored boxes and detailed information is shown with logos obtained from the MEME Suite website. The bit score is proportional to the frequency of the corresponding amino acid at each position.

The phylogenetic tree generated by cluster analysis classified the 25 *JrSWEETs* into four groups (Figure 3a). To better understand the genesis of the *JrSWEET* gene family, we analyzed the gene structure, including exon and intron distribution, examined the conserved regions and motifs, and predicted the transmembrane helices for each gene. We mapped gene structures using the GSDS (gene structure display server) and found that the coding regions of *JrSWEET* genes were interrupted with introns of varying number and size (Figure 3b). Most *JrSWEET* genes had five introns, with the exceptions that no introns were present in *JrSWEET1A* and *JrSWEET1B*, four in *JrSWEET4A*, six in *JrSWEET2* and *JrSWEET14A*, and seven in *JrSWEET17* (Figure 3b).

In addition to the nucleotide sequence analysis, the amino acid sequences of JrSWEETs revealed transmembrane helices and conserved motifs in the protein sequence (Figure 3c), as predicted by the MEME program. JrSWEET3A and JrSWEET16A encoded five transmembrane helices; JrSWEET4A, 17B, 14A, and 10A, six transmembrane helices; and the remaining JrSWEET family members encoded seven transmembrane helices. Five amino acid motifs were identified in all 25 JrSWEETs, all in the same order (motif 5, motif 1, motif 4, motif 2, and motif 3), except in JrSWEET16A, which lacked motifs 2 and 3, but contained an additional motif 5 (Figure 3d).

### 2.3. Phylogenetic Analysis of JrSWEET Proteins

**Figure 4:**
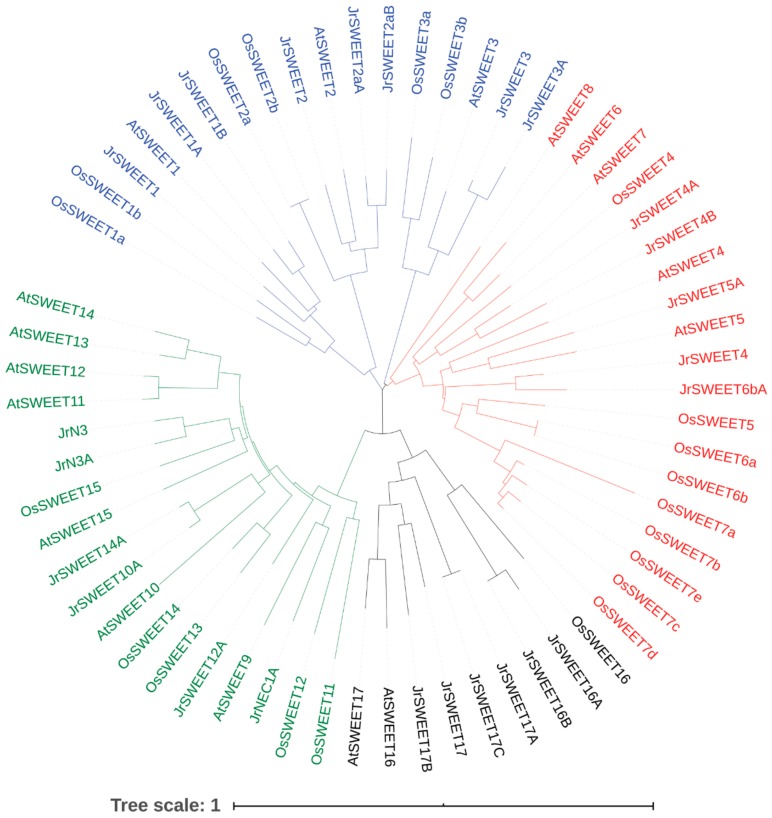
Unrooted neighbor-joining phylogenetic tree of SWEET proteins in *Arabidopsis*, rice, and walnut. Different colors represent the four different clades: blue, clade I; red, clade II; green, clade III; and black, clade IV. The scale bar represents 0.1 substitutions per amino acid position.

To better understand the evolutionary relationships among *SWEET* genes in walnut, *Arabidopsis*, and rice, the 25 JrSWEET proteins, 17 AtSWEET proteins, and 21 OsSWEET proteins were aligned by ClustalX and used to construct an unrooted phylogenetic tree using neighbor-joining implemented in MEGA 7.0. All 63 SWEET proteins fell into four clades. Clade I, clade II, and clade III contained 17, 19, and 18 proteins, respectively, while clade IV had only nine. Among the JrSWEET proteins, clade I contained eight members, followed by clades III and IV with six each, and clade II with only five. Walnut had more proteins in clade IV and fewer in clade II than *Arabidopsis* and rice (Figure 4).

### 2.4. Expression Profiles of JrSWEETs in Different Tissues and Organs and at Different Developmental Stages

**Figure 5:**
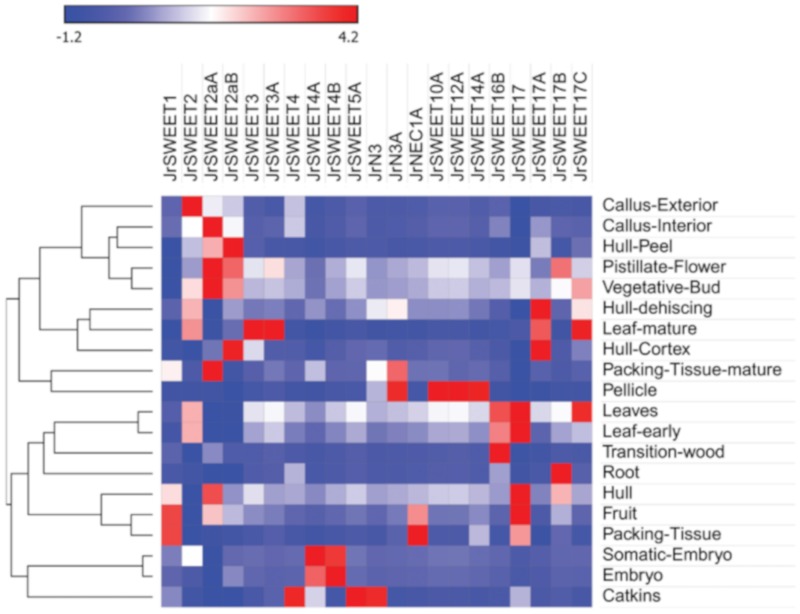
Hierarchical clustering and heat map of *JrSWEET* genes expressed in different tissues. The FPKM values obtained from RNA-sequencing were used to represent relative expression. Data shown in the heat map was standardized by Z-score.

To understand the roles of JrSWEETs during plant growth and development, expression patterns of the 25 *JrSWEET*s in different tissues and at different developmental stages were extracted from the 20 library walnut RNA-seq dataset [39,40]. The expression of four genes, namely *JrSWEET1A*, *JrSWEET1B*, *JrSWEET6bA*, and *JrSWEET16A*, was either very low or undetectable, which suggests that they may be expressed in highly specific tissues and/or under specific conditions. Transcriptomic analysis revealed that the other 21 genes, including orthologs, had varied expression patterns in different tissues and at different developmental stages (Figure 5). In clade I, *JrSWEET1* was highly expressed in fruit and packing tissues. *JrSWEET2* and orthologs *JrSWEET2aA* and *JrSWEET2aB* were expressed in most tissues and highly expressed in the callus, but *JrSWEET2* was present in the callus-exterior, *JrSWEET2aA* was present in the callus-interior, pistillate flower, vegetative bud, and mature packing tissue, while *JrSWEET2aB* was mainly present in hull peel and hull cortex. *JrSWEET3* and its paralog, *JrSWEET3A*, both had high expression in mature leaf. Two genes from clade II, namely *JrSWEET4* and *JrSWEET5aA*, were highly expressed in catkins, while the other genes of clade II, namely orthologs *JrSWEET4A* and *JrSWEET4B*, were both expressed most in somatic embryo and zygotic embryo. In clade III, *JrSWEET10A*, *JrSWEET12A*, and *JrSWEET14A* all displayed uniquely strong expression in the pellicle, *JrN3A* (an ortholog of *AtSWEET15*) showed strong expression both in mature packing tissue and pellicle, but JrN3, the paralog of *JrN3A*, was strongly expressed in catkins. *JrNEC1A* (an ortholog of *AtSWEET9*) was expressed most strongly in packing tissue and fruit. In clade IV, the five expressed genes were differentially expressed in all tissues. Among them, *JrSWEET16B* was highly expressed in transition wood, leaves, and early leaves. *JrSWEET17B* had the greatest expression in root. *JrSWEET17* was highly expressed in many tissues, including leaves, early leaves, hull, and fruit. Orthologs *JrSWEET17A* and *JrSWEET17B* both were highly expressed in dehiscing hull and mature leaf, but *JrSWEET17A* also had strong expression in hull cortex, while *JrSWEET17B* was highly expressed in vegetative buds and leaves. These results suggest that all *JrSWEET*s have differential expression in different tissues, which may contribute to the functional diversity of *JrSWEET*s.

**Figure 6:**
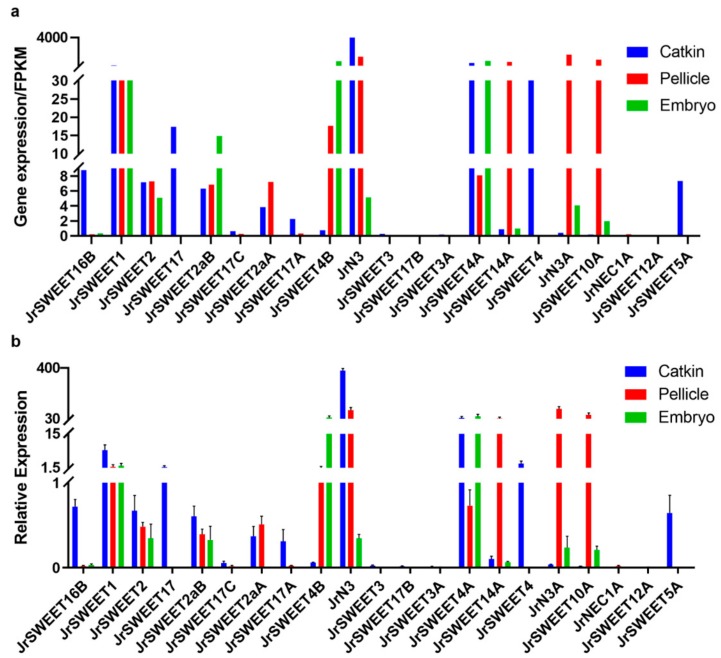
Expression of *JrSWEET* genes in catkin, pellicle, and embryo tissues. (**a**) Gene expression in three tissues, with FPKM values obtained from RNA-seq libraries containing five replicas of each tissue type. (**b**) Relative gene expression compared to *Jr18S*-11 in three tissues, with expression data obtained by qRT-PCR. Mean values and error bars were calculated from triplicate measurements.

Expression of *JrSWEET* genes was further validated by qRT-PCR in catkins, pellicle, and embryo tissues (Figure 6). Expression of 21 *JrSWEET* genes was detected by qRT-PCR, with almost the same expression patterns and profiles obtained from RNA-seq data except for *JrSWEET2aB*, which was highly expressed in catkins instead of pellicle. In addition, *JrSWEET16B*, *JrSWEET17*, *JrSWEET4*, and *JrSWEET5A* exhibited a unique high expression in catkins, while *JrSWEET14A*, *JrN3A*, and *JrSWEET10A* were expressed in pellicle. The expression pattern was clearly different for individual clade members and further functional analysis of *JrSWEET* genes is necessary to define more clearly the roles of individual members of the family.

### 2.5. Expression Analysis of JrSWEET Genes after Xaj417 Infection

**Figure 7:**
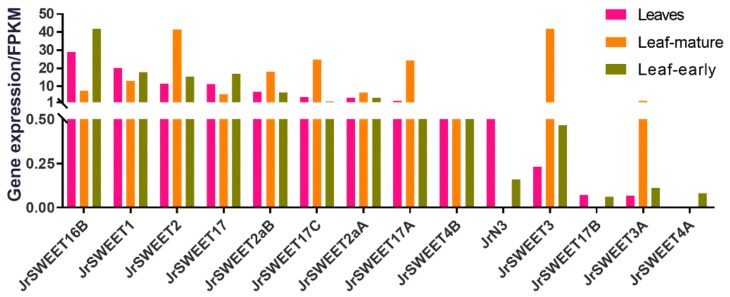
Expression of *JrSWEET* genes in leaves, leaf-mature, and leaf-early, with FPKM values obtained from RNA-sequencing. Expression of 21 of the 25 *JrSWEET* genes was detected in the complete 20 library RNA-seq data, but only 14 were expressed in at least one of the 3 leaf libraries analyzed.

**Figure 8:**
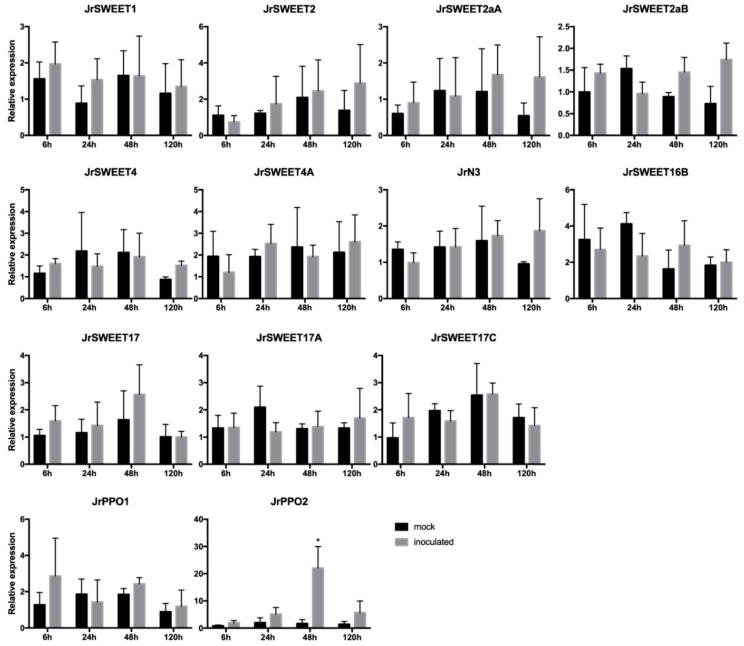
Relative expression of 11 *JrSWEET* genes detected at different times after inoculation with *Xaj*417. The other 14 *JrSWEET* genes were not detected (not shown). Mock treatments received water, and inoculated treatments were infected with *Xaj*417 bacteria. Relative gene expression in mock and inoculated tissues was measured at 0, 24, 48, and 120 h post inoculation. Asterisks indicate a significant difference (*p* < 0.05 in multiple *T* tests) in expression between mock and inoculated treatments, as seen for *JrPPO2* used as a reference for responsive genes.

To better understand the response of *JrSWEET* genes to *Xaj*, qRT-PCR was used to examine the differences in expression of individual *JrSWEET* genes at different times after infection with *Xaj*417. As a base line for these studies, we initially examined the expression pattern in the RNA-seq data of *JrSWEET* genes in leaf tissues that showed expression for 14 of them (Figure 7). As the original RNA-seq libraries did not contain tissues infected with *Xaj*, in a following set of experiments walnut microshoots were inoculated with *Xaj*417 and gene expression of *JrSWEET* genes analyzed by qRT-PCR. In this set, expression of only 11 *JrSWEET*s was detected (Figure 8). Most *JrSWEET* genes detected in one dataset was also detected in the other. The exceptions were *JrSWEET3* and *JrSWEET3A*, which were highly expressed in mature leaves in the RNA-seq data, but undetectable by qRT-PCR on leaves of *Xaj*-infected microshoots. Different types of leaf tissues were examined in the two datasets, which could explain the difference in expression patterns. The RNA-seq data was obtained from mature leaves harvested from trees in the field, while the inoculated tissues were leaves obtained from in vitro-grown shoots. *JrSWEET4A* was detected in in vitro shoots, but not in leaves from field grown trees, although the RNA-seq dataset showed extremely high expression in catkins and embryo.

**Figure 9:**
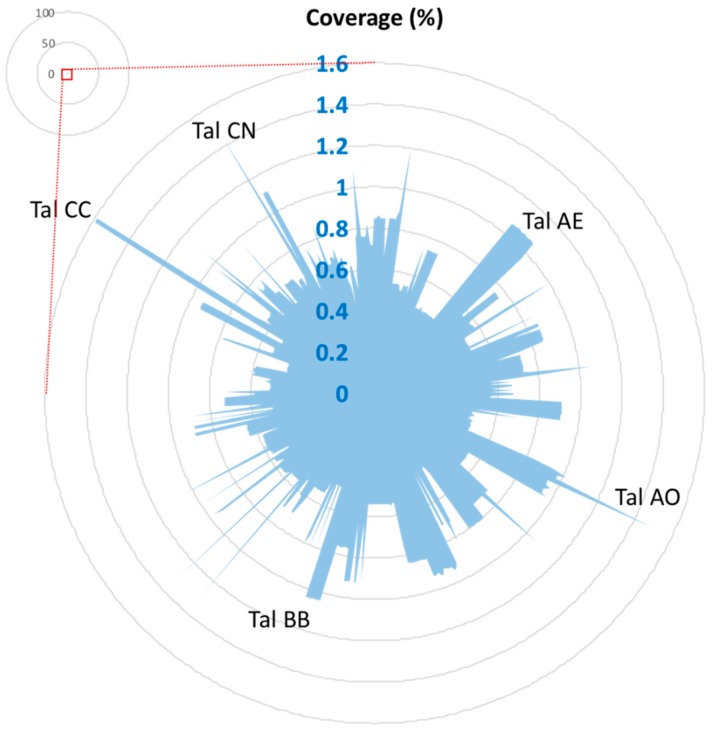
Blast results of known transcription activator-like effectors (TALEs) to the *Xaj*417 genome. Note the low percentage coverage of each 516 putative reported TALE in the *Xaj*417 genome (shown in blue).

Unexpectedly, no *JrSWEET* genes were induced by *Xaj* when assayed by qRT-PCR, although *JrPPO2* showed a 20.2-fold upregulation at 48 h post inoculation (Figure 8). To further examine this lack of *JrSWEET* response to *Xaj*417 infection, we analyzed the genome of the pathogen to identify candidate TALEs in *Xaj* that could possibly bind to the 5′ regulatory region of one or more *JrSWEET* genes. Interestingly, the results from the AnnoTALE search [41] showed that there were no TALEs in *Xaj*. Further confirmation was obtained by comparing all 516 known TALEs to the *Xaj*417 genome via BLAST. None displayed sequence coverage greater than 2% except for TalAE/AO/BB/CC/CN, confirming that there is no sequence in the *Xaj*417 genome with enough coverage and high identity that could be a genuine TALE homolog (Figure 9, Supplementary Materials Table S1). The lack of TALEs in *Xaj* may be connected to the reason why no *JrSWEET* genes were induced by *Xaj* and confirm there is no TALE–*SWEET* interactions during walnut blight development.

### 2.6. Prediction and Functional Analysis of Cis-Regulatory Elements in Putative Promoter Regions

**Figure 10:**
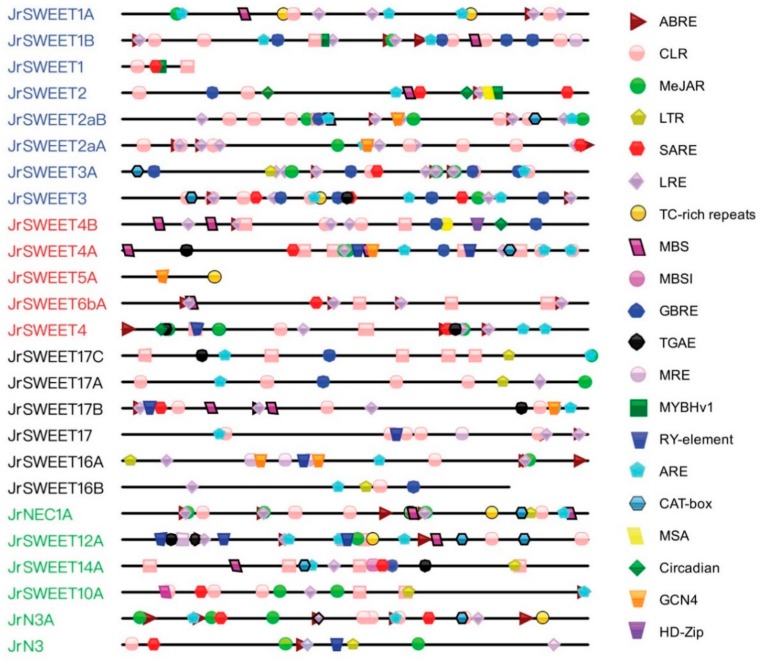
Predicted *cis*-elements responding to plant growth regulation, hormone response, and stresses response present in *JrSWEET* 5′ regulatory regions. The *cis*-elements are identified by function: ABRE, *cis*-acting element involved in abscisic acid responsiveness; CLR, part of a conserved DNA module involved in light responsiveness; MeJAR, *cis*-acting regulatory element involved in MeJA (methyl jasmonic acid)-responsiveness; LTR, *cis*-acting element involved in low-temperature responsiveness; SARE, *cis*-acting element involved in salicylic acid responsiveness; LRE, *cis*-acting regulatory element involved in light responsiveness; TC-rich repeats, *cis*-acting element involved in defense and stress responsiveness; MBS, MYB binding site involved in drought-inducibility; MBSI, MYB binding site involved in flavonoid biosynthetic gene regulation; GBRE, gibberellin-responsive element; TGAE, auxin-responsive element; MRE, MYB binding site involved in light responsiveness; MYBHv1, MYBHv1 binding site; RY-element, *cis*-acting regulatory element involved in seed-specific regulation; ARE, *cis*-acting regulatory element essential for anaerobic induction; CAT-box, *cis*-acting regulatory element related to meristem expression; MSA, *cis*-acting element involved in cell cycle regulation; Circadian, *cis*-acting regulatory element involved in circadian control; GCN4, *cis*-regulatory element involved in endosperm expression; and HD-Zip, element involved in differentiation of the palisade mesophyll cells.

To further understand the transcriptional regulation and potential functions of JrSWEETs, 5′ regulatory elements in the 2000 bp sequences upstream of the translation start sites were determined and analyzed on the PlantCARE server [42]. For *JrSWEET1*, *JrSWEET5A*, and *JrSWEET16B*, only smaller fragments could be obtained from the databases due to the short intergenic space between genes. Twenty different types of *cis*-regulatory elements were detected in 5′ regulatory regions of *JrSWEET* genes. These included light response (elements involved in light responsiveness, light response element, MYB binding sites), stress response (low temperature, drought, wound, anaerobic induction, defense, and stress), hormone responsive (auxin, abscisic acid, gibberellic acid, jasmonic acid, and salicylic acid), and growth and development regulation elements (seed-specific regulation, meristem expression, cell cycle regulation, circadian control, endosperm expression, and palisade mesophyll cells elements). Each gene contained from two to twenty-four elements (Figure 10). The most frequent elements were light response elements. All 25 *JrSWEET* 5′ regulatory regions contained at least one light responsive element except for *JrSWEET5A*. Secondly, 76%, 72%, and 64% of *JrSWEET* 5′ regulatory regions contained elements related to anaerobic induction, abscisic acid, and jasmonic acid responsiveness, respectively. MYB binding sites mostly concentrated in clade I, but appeared twice separately in clades IV and III, with none in clade II. Similarly, low-temperature responsiveness was absent from clade II; elements related to endosperm expression, from clade III; elements related to meristem expression, defense and stress, from clade IV; and auxin responsive elements, from clade I. Salicylic acid responsiveness, drought inducibility elements, gibberellic acid responsiveness, and seed-specific regulation elements occurred randomly in all clades. Only the 5′ regulatory region of *JrSWEET4B* contained an element related to palisade mesophyll cells; *JrSWEET2* and *JrSWEET4B* contained an element related to circadian control; and *JrSWEET2*, *JrSWEET4B*, and *JrSWEET4* contained elements related to cell cycle regulation. These data indicate that *JrSWEET* clades may have specialization, and further analysis is necessary to understand the function of each *SWEET* gene.

## 3. Discussion

Sugar transporters play a key role in growth and development of many plant species [34,43,44]. SWEETs are critically important for sugar efflux, phloem loading, and nectar secretion [11,45]. These transporters are also important susceptibility genes facilitating interaction between host plants and *Xanthomonas* pathogens [21,22]. Once secreted into host cells through T3SS, TALEs bind to the promoter region of specific sugar transporter genes favoring bacterial growth [20]. Different *Xanthomonas* strains have different repertoires of effector protein including TALEs that are important for virulence in several plant diseases [36]. Distribution of TALEs varies in *Xanthomonas* species depending on host specificity and geographic origin [46]. *X. oryzae* pathogens typically harbor large TALE repertoires [47], followed by *X. campestris* [48]. Interestingly, among *X. arboricola*, only pathovar *corylina* presents a TALE homolog [49]. To better understand the susceptibility of walnut to *Xaj*, the causative agent of walnut blight disease, we characterized the *SWEET* gene family in walnut as a potential source of susceptibility and their response to *Xaj*417 infection. This study represents the first detailed characterization of the SWEET gene family in a woody plant.

Twenty-five *SWEET* genes were detected in the *J. regia* genome, all highly conserved to their respective orthologs in other plants. All contained two MtN3 units and five motifs except *JrSWEET16A*, which had only four motifs. In general, the number of *SWEET* genes is highly variable within the plant kingdom, ranging from one to three copies in green algae to 18 to 68 in vascular plants [34]. Comparing the set of 25 *SWEET* genes in walnut to 17 in *Arabidopsis* suggests the possibility of functional redundancy or diversification among *SWEET* gene family members. It also implies that *SWEET* genes expanded during the course of evolution. In this study, five pairs of *JrSWEET* genes were detected as tandem duplications. This finding agrees with other reports of *SWEET* gene duplications, including segmental, tandem, or whole genome duplications [32,33,50]. However, gene loss during expansion was also found in the *J. regia* genome, compared to the *AtSWEET* and *OsSWEET* genes. There was no homolog of *AtSWEET7*, *AtSWEET8*, *AtSWEET11*, *AtSWEET13*, or *AtSWEET18* in the *J. regia* genome. All *AtSWEET* genes in clade III except *AtSWEET15* (ortholog of *JrN3* and *JrN3A*), *AtSWEET5*, and *AtSWEET6* have only one respective orthologous gene in the *J. regia* genome [28]. Expansion and/or loss of *SWEET* genes have also occurred in other species, indicating possible functional variation in the evolution of this gene family. For example, the absence of *SWEET10* in rice and wheat may suggest different roles of *SWEET* genes in dicot and monocot plants [15,32]. The expansion of *SWEET* genes in cucumber suggests possible functional differentiations in response to environmental conditions [51]. Additionally, five transmembrane helixes (TMHs) were observed in JrSWEET3A, JrSWEET16A, JrSWEET10A, and JrSWEET14A or six in JrSWEET4A and JrSWEET17B, which is less than the seven TMHs in eukaryotic SWEETs reported in previous studies. Gene loss or expansion and occurrence of some SWEETs with only five or six TMHs imply that duplication and fusion of SWEETs may be ongoing in the walnut genome. Our results support the proposal that the SWEET protein family was generated through duplication and fusion of SemiSWEETs, which contain three TMHs [12], rather than fusion of an archaeal with a bacterial SemiSWEET [52].

Interestingly, there was significant variation among SWEET proteins in length, predicted isoelectric points, and classification into four clades. This indicates that different SWEET proteins may function in different microenvironments, consistent with previous studies of SWEET functions [43]. Due to their functional diversity, we examined expression patterns of *JrSWEET* genes in different tissues. Expression of four *JrSWEET* genes was undetectable in any tissues/organs. This suggests that these genes may have degenerated or lost function after gene duplication during evolution. The expression patterns of the other *SWEET* genes were diverse among subfamilies, members of the same clade, and even between orthologous genes. The expression pattern of the *SWEET* gene family in sweet orange, banana, and other species suggests that SWEETs may undergo functionalization in many higher plant species [33,53,54]. Consistent with expectations, *JrSWEET* genes were expressed widely in all tested tissues, implying functional redundancy and an important role in both vegetative and reproductive growth. *JrSWEET* genes from clade I varied greatly among members. *JrSWEET1* was strongly expressed in fruits and packing-tissues, indicating it may regulate sugar allocation during fruit ripening by functioning as a bidirectional and low-affinity glucose transporter. However, expression of *JrSWEET2* and its orthologous genes in a variety of tissues, especially callus, indicates an important role in differentiation and development. *SWEET* genes from clade II generally function as bidirectional, low-affinity glucose transporters and are expressed mainly in floral organs [50,51,52]. *JrSWEET4*, *JrSWEET5*, and their orthologs are strongly expressed in catkins and embryos, providing evidence that they may play a role in reproductive development. *AtSWEET5* is expressed at different stages of pollen development [55], *AtSWEET8* is strongly expressed in microspores and tapetum during male meiosis, and *AtSWEET13* can partially complement the defective pollen phenotype at later reproductive stages, indicating a broader role for SWEETs in pollen maturation [56,57]. This also explains the strong expression of *JrN3* in catkins. Clade IV members are strongly expressed in root cortex and encode proteins that function as fructose-specific uniporters in the root tonoplast [58]. This explains the specific and strong expression of *JrSWEET17B* in root. Most of the 25 *JrSWEET* genes were predicted on the basis of structure to be localized in the plasma membrane, except for JrSWEET2aB and JrSWEET17, which are present in the vacuolar membrane, and JrSWEET17A, present in the chloroplast. These were validated by differential expression in different tissues, further suggesting that *JrSWEET* genes have developed a diversity of roles for sugar transport.

Members of clade III SWEET genes transport sucrose, which likely serves as a carbon source for plant pathogens, and show highly induced expression as sugar transporters during disease development. Several studies report involvement of *SWEET* genes in various plant pathogenic systems [20,21,22,23,25,26]. *OsSWEET11*, *OsSWEET12*, *OsSWEET13*, *OsSWEET14*, and their orthologs are susceptibility genes in rice infected by *Xoo*. *MeSWEET10a* in the dicot cassava is induced by infection with *X. axonopodis*, and *CsSWEET1* in citrus is induced by infection with *Xcc*. *SWEET* genes are also induced by other bacterial and fungal pathogens, suggesting that *SWEET* induction may be a frequent strategy adopted by diverse pathogens to steal sugar from plants. In walnut, most *JrSWEET* genes in clade III are highly expressed in pellicle, a tissue typically attacked by microorganisms. We detected expression of 11 *SWEET* genes in microshoots’ leaves during infection by *Xaj*417, but interestingly, none showed increased expression in response to infection. Considering that this particular pathogen strain has a functional type III secretion system that in other *Xanthomonas* pathogens is able to secrete TALEs into the plant cell and influence *SWEET* genes expression [59], a logical hypothesis was that *Xaj*417 would adopt this strategy. If this was the case, a disease control strategy could encompass the inactivation of a particular responsive *SWEET* member in walnut to deprive the pathogen from this sugar source, as recently shown for the rice blight pathosystem [60,61]. Since we observed no induction of specific *SWEET* genes in response to *Xaj*417 infection, we investigated the pathogen’s genome in search of any known TALEs. Surprisingly, none were found, which may be connected with the observed lack of *SWEET* gene induction in response to *Xaj*417 infection. On the other hand, it cannot be excluded that a significant response would be detected at other time points or conditions (e.g., pot-experiment), although at up to 168 hpi no induction was observed (data not shown). Our results suggest that *SWEET* genes do not play a critical role in walnut blight susceptibility, and the absence of TALEs in *Xaj*417 indicates some other infection strategies are the source of susceptibility to walnut blight disease. The type II secretion system of *Xaj*417, for example, may play a greater role in increasing susceptibility to walnut blight disease [62].

We identified and characterized 25 *SWEET* genes in walnut, using their unique DNA sequences to determine a variety of parameters such as genomic location, predicted number of amino acids (length) and molecular weight, isoelectric point, sub-cellular localization, exon–intron structures and conserved motifs, phylogeny, expression analysis, and presence of *cis*-elements. The results from this study provide a basic understanding of the *JrSWEET* genes, facilitate further detailed research on *SWEET* genes in walnut, and provide a platform for identification and comprehensive functional characterization of *SWEET* gene families from other woody plants. The non-participation of *SWEET* genes in increasing susceptibility to walnut blight, along with the observation that no TALEs are present in the *Xaj*417 genome, suggest that other avenues of research into plant–pathogen interactions other than those mediated by TALE–SWEET genes may be more fruitful in developing strategies to improve resistance to walnut blight.

## 4. Materials and Methods

### 4.1. Genome-Wide Identification of SWEET Family Genes in Walnut

To identify walnut *SWEET* genes, two different approaches were used to compile a complete list. We downloaded the latest version of the whole *Juglans regia* genome annotation from the National Center for Biotechnology Information (NCBI). In one approach, we obtained putative *SWEET* genes by searching for the Hidden Markov Models (HMMER. http://hmmer.org) profile of the core domain MtN3/saliva protein (PF03083) from the Pfam database (Pfam. http://pfam.xfam.org) [63,64]. The other approach was to retrieve all 17 known SWEET proteins in *A. thaliana* from the *Arabidopsis* Information Resource (TAIR. https://www.arabidopsis.org/). Putative walnut SWEET proteins were identified by blast searches against the walnut genome using *A. thaliana* SWEET protein sequences as queries. In addition, we used “bidirectional sugar transporter” as a query keyword to search all walnut paralog genes listed in the Kyoto Encyclopedia of Genes and Genomes database (KEGG. https://www.genome.jp/kegg/) [65]. To confirm the core domain and completeness, all candidate proteins were verified manually with the Conserved Domain Database (CDD, https://www.ncbi.nlm.nih.gov/cdd) and SMART (SMART. http://smart.embl-heidelberg.de/) [66,67]. Some candidates were abandoned as too short or incomplete. The remaining proteins were reconfirmed by length of sequence, isoelectric point (pI), and molecular weight (Mw) using the ExPASy tool (ExPASy. https://web.expasy.org/compute_pi/) [68]. The subcellular localization of JrSWEET proteins was predicted through the TargetP 1.1 server (TargetP. http://www.cbs.dtu.dk/services/TargetP/) and WoLF PSORT (WoLF PSORT. https://wolfpsort.hgc.jp/) [69,70]. Moreover, we recovered the locational information of every single putative gene from the walnut genome databases (http://aegilops.wheat.ucdavis.edu/Walnut/data.php) and sketched all *SWEET* genes onto their respective chromosomes using Microsoft PowerPoint 2017 [71].

### 4.2. Gene Structure Analysis, Transmembrane Helix, and Motif Recognition of JrSWEETs

Information on untranslated regions (UTR), exons, and introns were obtained from NCBI by aligning the cDNA sequences with their corresponding mRNA sequence or genomic DNA sequences. Identification of predicted transmembrane helices in proteins was by TMHMM Server 2.0 (TMHMM. http://www.cbs.dtu.dk/services/TMHMM/) [72]. The gene and protein structures were constructed using the Gene Structure Display Server (GSDS. http://gsds.cbi.pku.edu.cn) [73]. The phylogenetic tree of walnut *SWEET* family members was constructed using unrooted neighbor-joining analysis in MEGA 7.0 (MEGA. https://www.megasoftware.net/), with bootstrap tests carried out 1000 times. The conserved motifs in SWEET proteins and the logos of motifs were generated by the MEME suite (MEME. http://meme-suite.org) and redrawn by TBtools [74].

### 4.3. Phylogenetic Analysis of JrSWEET Proteins

The full-length amino acid sequences of 17 *AtSWEET* genes derived from TAIR and 21 *OsSWEET* genes from the rice genome database in the rice genome annotation project (TIGR. http://rice.tigr.org) were combined with newly identified *JrSWEET* genes for phylogenetic analysis. All acquired protein sequences were first aligned by ClustalW with default parameters, then an unrooted neighbor-joining analysis was used to construct the phylogenetic tree using MEGA 7.0 with bootstrap test carried out 1000 times. The tree was further edited by iTOL (iTOL. https://itol.embl.de) [75].

### 4.4. Expression Profiles of JrSWEETs in Different Tissues, Organs, and Developmental Stages from RNA-Seq Data

Expression data of *JrSWEET* transcripts was obtained from RNA-seq data for twenty different tissues and/or developmental stages of ‘Chandler’ walnut [76]. Transcript abundance of 25 *JrSWEET* genes in thirteen tissues (callus, catkins, zygotic embryo, pistillate flower, hull, fruit, leaves, packing tissue, pellicle, root, somatic embryo, wood, and bud) and developmental stages of five tissues (callus, hull, leaf, packing tissue, and zygotic embryo) was analyzed. The expression data was used to generate a heat map using the Morpheus heat map program (MORPHEUS. https://software.broadinstitute.org/morpheus/) package with hierarchical clustering for twenty different tissues.

### 4.5. Expression of JrSWEET Genes after Inoculation with Xaj417

Tissue-cultured ‘Chandler’ walnut microshoots were transferred to fresh medium in individual culture tubes two days prior to inoculation with *Xaj*417, a copper-resistant strain isolated from diseased vegetative buds in California and sequenced at the University of California, Davis [77]. A frozen stock culture (stored in 60% glycerol at −80 °C) was grown on yeast extract peptone (YEP) agar for 48 h and then transferred to liquid culture for 16 h. Bacterial suspensions were diluted in 0.01% (*v*/*v*) Tween 80 solution to ~2 × 10^8^ CFU/mL using a spectrophotometer at 600 nm, then diluted to 2 × 10^7^ CFU/mL for inoculation. To inoculate the microshoots, 30 mL of bacterial suspension was poured into each tube, and whole microshoots were immersed for 20 min in the inoculum and then drained for an additional 10 min. The mock inoculation used 0.01% (*v*/*v*) Tween 80 solution instead of bacterial suspension. Forty-eight inoculated shoots were grown in individual tubes at 26 °C with a 16 h/8 h (light/dark) photoperiod. A leaf was harvested from each shoot at the time of inoculation, then another leaf was harvested from each of three shoot replicates at 6, 24, 48, and 120 h after inoculation per treatment (time points selected according to previous experiments). Harvested leaves were frozen immediately in liquid nitrogen and stored at −80 °C until use. All inoculation experiments were repeated at least three times.

### 4.6. Total RNA Isolation and Gene Expression by Quantitative Real-Time PCR (qRT-PCR)

Pellicles, catkins, and mature zygotic embryos were obtained from a field-grown ‘Chandler’ tree located at Davis, CA. Roots were obtained from embryos grown in tissue culture. Embryos were allowed to germinate, and leaves were harvested for gene expression analysis after inoculation with *Xaj*417. After collection, all tissues were frozen immediately in liquid nitrogen and stored at −80 °C until use. Total RNA was extracted using Plant RNA Purification Reagent (Thermo Fisher Scientific) and purified using the RNeasy MinElute Clean-up Kit (Qiagen, Hilden, Germany) following the manufacturer’s instructions. The quality of RNA was determined using a nanodrop and also by agarose gel electrophoresis. Complementary DNA (cDNA) was synthesized using a QuantiTect Reverse Transcription Kit (Qiagen) according to the manufacturer’s instructions. Quantitative real-time PCR was performed with SYBR Green PCR Master Mix (Applied Biosystems, Foster City, CA, USA) using an ABI StepOne Real-Time PCR system and software (Applied Biosystems). The primers used for each gene analyzed are listed in Supplementary Table S2. The protocol was initiated with a step of 10 s at 95 °C, followed by 40 cycles of 95 °C for 10 s and 62 °C for 30 s, ending with a melting curve that was used to verify the specificity of each reaction. All reactions for each gene, including negative controls (water instead of template), were performed in triplicate. All gene expression bar graphs were draw by Prism 7.0 software package with multiple *T* tests for significance analysis (*p* < 0.05) [78].

### 4.7. Searching for TALEs in Xaj417

To seek potential target *JrSWEET* genes through predicted TALE binding sites, we used three ways to identify TALEs in the whole *Xaj*417 genome downloaded from NCBI (https://www.ncbi.nlm.nih.gov/genome/11823?genome_assembly_id=244764). First, we used AnnoTALE to search for TALEs in the whole genome [41]. Next, all known TALEs and their sequences in *Xanthomonas* that were listed at The *Xanthomonas* Resource (http://www.xanthomonas.org/tools.html) were downloaded. Finally, a blast search using all 516 TALEs in The Xanthomonas Resource was performed against the *Xanthomonas arboricola* pv. *juglandis* strain 417 genome.

### 4.8. Prediction and Functional Analysis of Cis-Regulatory Elements in Putative Promoter Regions

The 2000 bp genomic DNA sequences upstream of the translation start sites of the *JrSWEET* genes were searched for 5′ regulatory sequences using PlantCARE (PlantCARE. http://bioinformatics.psb.ugent.be/webtools/plantcare/html/) to identify *cis*-acting elements [42]. Twenty regulatory elements involved in plant growth regulation, hormone response, and stress responses were identified in the 5′ regulatory regions and diagrammed in Figure 10 using Illustrator for Biological Sequences (IBS. http://ibs.biocuckoo.org/) [79].

## Figures and Tables

**Table 1:** Description of walnut *SWEET* genes.

Clade	Gene Name	Locus	Size (aa) ^a^	Mw (kDa)	pI	Genomic Location	MtN3/Saliva (PQ-Loop Repeat) Domain Position	Loc ^b^
I	*JrSWEET1*	108999697	254	28.22	9.43	NW_017444989.1:303-3465	7–94, 131–213	PM
I	*JrSWEET1A*	109004166	258	28.22	6.3	NW_017388835.1:280022-280798	6–95, 129–215	PM
I	*JrSWEET1B*	109004173	261	29.01	9.35	NW_017388835.1:318614-319399	7–96, 130–216	PM
I	*JrSWEET2*	108985341	235	26.11	9.27	NW_017442681.1:95892-99748	18–102, 139–222	PM
I	*JrSWEET2aA*	108987476	235	25.72	8.84	NW_017442906.1:10127-13031	18–102, 138–219	PM
I	*JrSWEET2aB*	108998082	235	26.01	9.34	NW_017443613.1:377035-380456	18–102, 138–220	VM
I	*JrSWEET3*	108985064	246	27.72	9.15	NW_017442611.1:20354-22484	9–95, 133–216	PM
I	*JrSWEET3A*	108982726	292	33.24	9.32	NW_017441679.1:10593-14236	9–95, 121–201	PM
II	*JrSWEET4*	108997137	241	26.75	9.01	NW_017388976.1:192220-194338	10–97, 136–217	PM
II	*JrSWEET4A*	109002076	265	29.06	9.13	NW_017443554.1:481695-483478	10–97, 133–217	PM
II	*JrSWEET4B*	108993759	301	33.19	9.23	NW_017443600.1:567007-569556	49–136, 172–256	PM
II	*JrSWEET5A*	108999161	231	26.37	8.9	NW_017443629.1:533788-536495	10–95, 133–216	PM
II	*JrSWEET6bA*	108992796	225	24.89	9.3	NW_017443540.1:669544-671368	9–98, 134–220	PM
III	*JrN3*	108984368	295	33.08	7.63	NW_017442395.1:48585-51197	12–96, 132–217	PM
III	*JrN3A*	109004781	294	32.77	8.29	NW_017389221.1:55377-57616	13–97, 133–219	PM
III	*JrNEC1A*	108992134	294	32.72	6.99	NW_017443525.1:147356-149392	14–97, 136–219	PM
III	*JrSWEET10A*	108990746	281	31.88	9.06	NW_017443364.1:192481-194495	10–95, 131–215	PM
III	*JrSWEET12A*	108990744	298	32.84	8.27	NW_017443364.1:211540-213673	13–98, 134–218	PM
III	*JrSWEET14A*	108998489	272	30.98	9	NW_017388807.1:776356-778642	10–96, 132–216	PM
IV	*JrSWEET16A*	109006766	154	16.63	9.72	NW_017389361.1:1228957-1230806	6–93	PM
IV	*JrSWEET16B*	108979882	277	30.37	9.52	NW_017439469.1:3236-6113	7–90, 128–211	PM
IV	*JrSWEET17*	108994709	247	27.37	5.87	NW_017443571.1:1587292-1596052	6–95, 133–214	VM
IV	*JrSWEET17A*	108987463	259	28.83	6.59	NW_017442898.1:55816-57539	16–97, 136–218	CL
IV	*JrSWEET17B*	108987468	247	27.03	7.73	NW_017442898.1:62119-64578	7–90, 128–212	PM
IV	*JrSWEET17C*	109012696	253	28.11	5.85	NW_017389857.1:1048384-1050148	9–90, 129–211	PM

^a^ aa: length of the protein’s amino acid chain; pI: isoelectric point; and Mw: molecular weight in kDa. ^b^ The Loc is the most probable subcellular location predicted by WoLFSORT and TargetP. PM: plasma membrane; VM: vacuolar membrane; and CL: chloroplast.

## Supplementary Materials

Supplementary materials can be found at https://www.mdpi.com/1422-0067/21/4/1251/s1.

## Abbreviations

SWEET	Sugar will eventually be exported transporters
*Xaj*	*Xanthomonas arboricola* pv. *juglandis*
TALEs	Transcription activator-like effectors
SUTs	Sucrose transporters
TMDs	Transmembrane domains
TMHs	Transmembrane helixes
PPO2	Polyphenol oxidase
qRT-PCR	Quantitative real-time PCR
pI	Isoelectric point
